# Retraction Note to: Citrate stabilized Fe_3_O_4_/DMG modified carbon paste electrode for determination of octamethylcyclotetrasiloxane in blood plasma and urine samples of cement factory workers

**DOI:** 10.1186/s13065-022-00798-x

**Published:** 2022-02-21

**Authors:** Rashid Heidarimoghadam, Abbas Farmany

**Affiliations:** 1 Health Sciences Research Center and Department of Ergonomics, School of Public Health, Hamadan University of Medical Sciences, Hamadan, Iran; 2 Dental Research Center, Hamadan University of Medical Sciences, Hamadan, Iran

## Retraction Note to: BMC Chemistry (2020) 14:29 https://doi.org/10.1186/s13065-020-00681-7

The Editor has retracted this article. After publication, concerns were raised about the reproducibility of the work due to poorly reported methods and the authors’ inability to share sufficient raw data. Further investigation concluded that:

- the presentation of the findings is misleading and their impact is overstated, specifically with regards to the analysis of biological samples;
- the article lacks discussion of the limitations and shortcomings, including the lack of relevant control experiments;
- the statements and claims in the article are supported by incorrect or inappropriate references or by citing derivations of original work.

The Editor therefore no longer has confidence in the results and conclusions of this article.

Abbas Farmany does not agree to this retraction. Rashid Heidarimoghadam has not responded to any correspondence from the editor or publisher about this retraction.

